# A novel intronic *TCOF1* pathogenic variant in a Chinese family with Treacher Collins syndrome

**DOI:** 10.1186/s12920-024-01828-4

**Published:** 2024-03-18

**Authors:** Haojie Sun, Xinda Xu, Binjun Chen, Yanmei Wang, Jihan Lyu, Luo Guo, Yasheng Yuan, Dongdong Ren

**Affiliations:** 1 Department of Otorhinolaryngology, ENT Institute, Eye and ENT Hospital, Fudan University, Shanghai, China; 2 NHC Key Laboratory of Hearing Medicine, Fudan University, Shanghai, China; 3 Shanghai Auditory Medical Center, Shanghai, China; 4 Department of the Affiliated Eye and ENT Hospital, State Key Laboratory of Medical Neurobiology, ENT Institute and Otorhinolaryngology, Fudan University, No. 83, Fenyang Road, Shanghai, 200031 China

**Keywords:** Treacher Collins syndrome, *TCOF1*, Heterozygous variants, Whole-exome sequencing, Minigene, Ossicular chain malformation

## Abstract

**Background:**

Treacher Collins syndrome (TCS; OMIM 154500) is a craniofacial developmental disorder.

**Methods:**

To investigate the genetic features of a four-generation Chinese family with TCS, clinical examinations, hearing tests, computed tomography, whole-exome sequencing (WES), Sanger sequencing, reverse transcription (RT)-PCR, and the Minigene assay were performed.

**Results:**

The probands, an 11-year-old male and his cousin exhibited typical clinical manifestations of TCS including conductive hearing loss, downward slanting palpebral fissures, and mandibular hypoplasia. Computed tomography revealed bilateral fusion of the anterior and posterior stapedial crura and malformation of the long crura of the incus. WES of both patients revealed a novel heterozygous intronic variant, i.e., c.4342 + 5_4342 + 8delGTGA (NM_001371623.1) in *TCOF1*. Minigene expression analysis revealed that the c.4342 + 5_4342 + 8delGTGA variant in *TCOF1* caused a partial deletion of exon 24 (c.4115_4342del: p.Gly1373_Arg1448del), which was predicted to yield a truncated protein. The deletion was further confirmed via RT-PCR and sequencing of DNA from proband blood cells. A heterozygous variant in the *POLR1C* gene (NM_203290; exon6; c.525delG) was found almost co-segregated with the *TCOF1* pathogenic variant.

**Conclusions:**

In conclusion, we identified a heterozygous *TCOF1* splicing variant c.4342 + 5_4342 + 8delGTGA (splicing) in a Chinese TSC family with ossicular chain malformations and facial anomalies. Our findings broadened the spectrum of TCS variants and will facilitate diagnostics and prognostic predictions.

**Supplementary Information:**

The online version contains supplementary material available at 10.1186/s12920-024-01828-4.

## Background

Treacher Collins syndrome (TCS; OMIM 154500) is a craniofacial developmental disorder with an incidence of 1 in 50,000 live births [1]. TCS is characterized by malar and mandibulomaxillary hypoplasia and periorbital anomalies [2]. The most common clinical manifestations include symmetrical malar hypoplasia, micrognathia, antimongoloid slanting palpebral fissures, malformed ears, external ear canal deficits, and conductive hearing loss. These clinical symptoms vary from severe to unaffected among patients [3].

External and middle ear, but not inner ear, anomalies are apparent in patients with TCS [4]. Conductive hearing loss occurs in 83–92% of patients, mainly due to abnormalities of the ossicular chain (including irregular or absent auditory ossicles, fusion of the malleus and incus, and partial absence of stapes). The frequencies of atresia of the external ear canal and microtia are 68–71% and 10–77% respectively [5–7].

To date, four clinical subtypes of TCS have been described, categorized by their pathogenic genes (Supplemental Table 1): TCS1 (OMIM 154500) caused by *TCOF1* gene mutation, TCS2 (OMIM 1613717) caused by *POLR1D* gene mutation, TCS3 (OMIM 248390) caused by *POLR1C* gene mutation, and TCS4 (OMIM 618939) caused by *POLR1B* gene mutation [8]. TCS is inherited in both autosomal dominant (*TCOF1, POLR1D,* and *POLR1B*) and autosomal recessive (*POLR1C* and *POLR1D*) modes.

We investigated a four-generation Chinese family with two TCS patients exhibiting typical clinical features including antimongoloid, slanting palpebral fissures, and bilateral ossicular chain malformation. A heterozygous c.4342 + 5_4342 + 8delGTGA (NM_001371623.1; exon24) splice-site variant in *TCOF1* was detected by whole-exome sequencing (WES). Minigene assay showed that the variant disrupted mRNA splicing and caused a 228-bp loss of exon 24, which was confirmed by in vivo RT-PCR and sequencing. This study identified a novel TCS-associated variant, explain the functional effects of the new pathogenic *TCOF1* variant, and expand the gene mutation spectrum of TCS.

## Methods

### Subjects

This study was performed after written informed consent was obtained from the patients’ guardians, in line with the Declaration of Helsinki, and was approved by the Medical Ethics Committee of the Eye & ENT Hospital of Fudan University (approval no. ChiCTR2100042585). Peripheral blood samples and comprehensive clinical information were collected from the patients and all available family members. Pure tone audiometry (PTA), acoustic immittance, and temporal bone high-resolution computed tomography (HRCT) were conducted.

### Trio-based WES

#### DNA library preparation

Genomic DNA was extracted from peripheral blood using a QIAamp DNA Mini Kit (Qiagen, Shanghai, China) following the manufacturer’s instructions. DNA was quantified using the Nanodrop 2000 system (Thermal Fisher Scientific, Waltham, MA, USA). Genomic DNA (1–3 μg) was fragmented to an average of 150 bp using a S220 Focused Ultrasonicator (Covaris, Woburn, MA, USA). A DNA Sample Preparation Reagent Set (MyGenostics, Beijing, China) was used to prepare standard libraries. The steps included end repair, adapter ligation, PCR amplification, and sequencing using the DNBSEQ-T7 system.

#### Enrichment and sequencing of targeted genes

Amplified DNA of 23,000 genes was obtained using the GenCap capture kit (MyGenostics). The biotinylated 100-bp capture probes tiled all coding exons and the 50-bp flanking regions of all genes. Capture was performed following the manufacturer’s protocol. Briefly, a DNA library (500 ng) was mixed with Buffer BL and the GenCap gene panel probes (MyGenostics); the mixture was heated at 95 °C for 5 min and then at 65 °C for 5 min in a PCR device. Next, 19 μL of prewarmed (65 °C) Buffer HY (MyGenostics) was added and the mixture held at 65 °C with the PCR lid closed for 16–24 h; hybridization was then performed. Next, 50 μL of MyOne beads (Life Technologies, Carlsbad, CA, USA) were washed three times with 50 μL 1 × binding buffer and the beads were resuspended in 50 μL of 1 × binding buffer and the hybrid mixture. The beads were washed with WB1 buffer at room temperature for 15 min, and with WB3 buffer at 65 °C three times for 10 min each time. Bound DNA was eluted with buffer and amplified over 13 cycles using the following program: 95 °C for 4 min (1 cycle), 98 °C for 30 s, 65 °C for 30 s, and 72 °C for 30 s (13 cycles), and 72 °C for 5 min (1 cycle). The PCR products were purified using SPRI beads (Beckman Coulter, Brea, CA, USA) using the manufacturer’s protocol. The enriched libraries were sequenced on the DNBSEQ T7 platform; paired-reads of 150 bp were obtained.

#### Bioinformatics analysis

After sequencing, the raw data were saved in FASTQ format. Both MGI sequencing adapters and low-quality reads (< 80 bp) were filtered away using Cutadapt software (http://code.google.com/p/cutadapt/). Clean reads were mapped to the UCSC hg19 human reference genome using the BWA package implemented by Sentieon software (https://www.sentieon.com/). Duplicated reads were removed using the parameter driver of Sentieon software, which also corrected the bases; this ensured that the base quality of the reads of the final BAM file accurately reflected the probability of mismatches with the reference genome. The mapped reads were used to detect variations. SNP and InDel variants were detected by the parameter driver. Then, the data were transformed to VCF format. Variants were further annotated using ANNOVAR software (http://annovar.openbioinformatics.org/en/latest/) and searched for in multiple databases: 1,000 Genomes, ESP6500, dbSNP, EXAC, Inhouse (MyGenostics), and HGMD. The variants were predicted by SIFT, PolyPhen-2, MutationTaster, and GERP +  + CNVkit software (https://cnvkit.readthedocs.io/en/stable/) was used to predict copy number variants (CNVs).

#### Variants selected

Four steps were used to select potential pathogenic mutations for downstream analysis: the number of mutations reads exceeded five and the mutation ratio was ≥ 30%; mutations were removed if their frequencies exceeded 5% in the 1,000 g, ESP6500, and Inhouse databases; mutations were removed if they were in the InNormal database (MyGenostics); and synonymous mutations were removed when they were not in the HGMD database. The remaining mutations were subjected to further analysis.

#### Family co-segregation analysis

Mutations identified via WES were verified by Sanger sequencing. A pair of primers were used to sequence the potential pathogenic variant in TCOF1 gene:

- F: 5’- AAAGCAACATCACCCAGTGC -3’;
- R: 5’- ATGGGAGGAATGAGACCAGG -3’;

PCR involved initial denaturation at 98 °C for 2 min, 10 cycles of denaturation at 98 °C for 10 s, annealing at 65 °C for 30 s, extension at 72 °C for 10 s, 25 cycles of denaturation at 98 °C for 10 s, annealing at 55 °C for 30 s, extension at 72 °C for 10 s, and a final extension at 72 °C for 1 min. Purified PCR products were subjected to Sanger sequencing (Biosune, Shanghai, China) and the results were analyzed using DNASTAR (Madison, WI, USA) software.

### Minigene construction, transfection. and reverse transcription (RT)-PCR

In vitro minigene splicing assays were performed as described previously [9] using an amplified sequence with the c.4342 + 5_4342 + 8delGTGA variant from a healthy control genome, and using the following primers (thus including exon 23, a portion of intron 23, exon 24, intron 24, and exon 25):

- 5'-AAGCTTGGTACCGAGCTCGGATCCTCTCTCCTCTCAGGTTATATGACCCCTGG-3' and
- 5'-TGGGAGGAAGTTGGCCTCTCAAGTAATTGGGACTAC-3', 5'-GAGAGGCCAACTTCCTCCCAGCTCCAGACTCCATC-3' and
- 5'-TTAAACGGGCCCTCTAGACTCGAGCTTTTTCTTCTTTTTCTTCTGGGACGGTG-3'.

The amplified product was cloned into the *Bam*HI/*Xho*I doubly digested pMini-CopGFP vector (Hitrobio.tech, Beijing, China) using the ClonExpress II One Step Cloning Kit (Vazyme, Nanjing, China). Mutant minigene plasmids were constructed via site-directed mutagenesis using the following primers:

- 5'-ACAAGAGTGACCGCTTCTCCCAGCCCACCCCAA-3' and
- 5'-AGAAGCGGTCACTCTTGTCGGATTTCTTCTTCT-3'.

Verified wild-type and mutant minigene plasmids were transfected into the HEK293T cell line for 48 h. Total RNA was extracted using the TRIzol reagent (Cowin Biotech Co., Beijing, China), and cDNA synthesized (Vazyme; catalog no. R212-01). RT-PCR was performed using the following primers: 5'-GGCTAACTAGAGAACCCACTGCTTA-3' and 5'-CTTTTTCTTCTTTTTCTTCTGGGAC-3'.

The lengths of the amplified fragments and spliced forms were determined by agarose gel electrophoresis and Sanger sequencing, respectively.

### Quantitative RT-PCR of TCOF1 expression, and Sanger sequencing

RT-PCR and Sanger sequencing were used to determine whether the mutation affected splicing. Total RNA was extracted from samples stored at − 80ºC using a PAXgene Blood RNA Kit (Qiagen; catalog no. #762,174) according to the manufacturer’s recommendations. The Qubit 3.0 Fluorometer (Life Technologies) and Nanodrop One spectrophotometer (Thermo Fisher Scientific) were used to determine RNA concentration and quality, respectively. The Agilent 2100 Biological Analyzer (Agilent Technologies Inc., CA, USA) was used to evaluate RNA integrity. Briefly, 1 µg of RNA was reverse-transcribed using a Hifair II First Strand cDNA Synthesis Kit (gDNA Digester Plus; Yeasen Biotech, Shanghai, China; catalog no. 11121ES50). Primers binding to exons 23–26 of TCOF1 were used for RT-PCR of the cDNA-amplified region:

- F: 5’- GCAGGCATGTTGTCCCCTAA-3’;
- R: 5’- GTGCTGGTGCTCGTCATACA-3’;

The molecular weights of RT-PCR products were determined via agarose gel electrophoresis followed by Sanger sequencing after band elution from the gel and purification.

## Results

### Patient characteristics

**Fig. 1:**
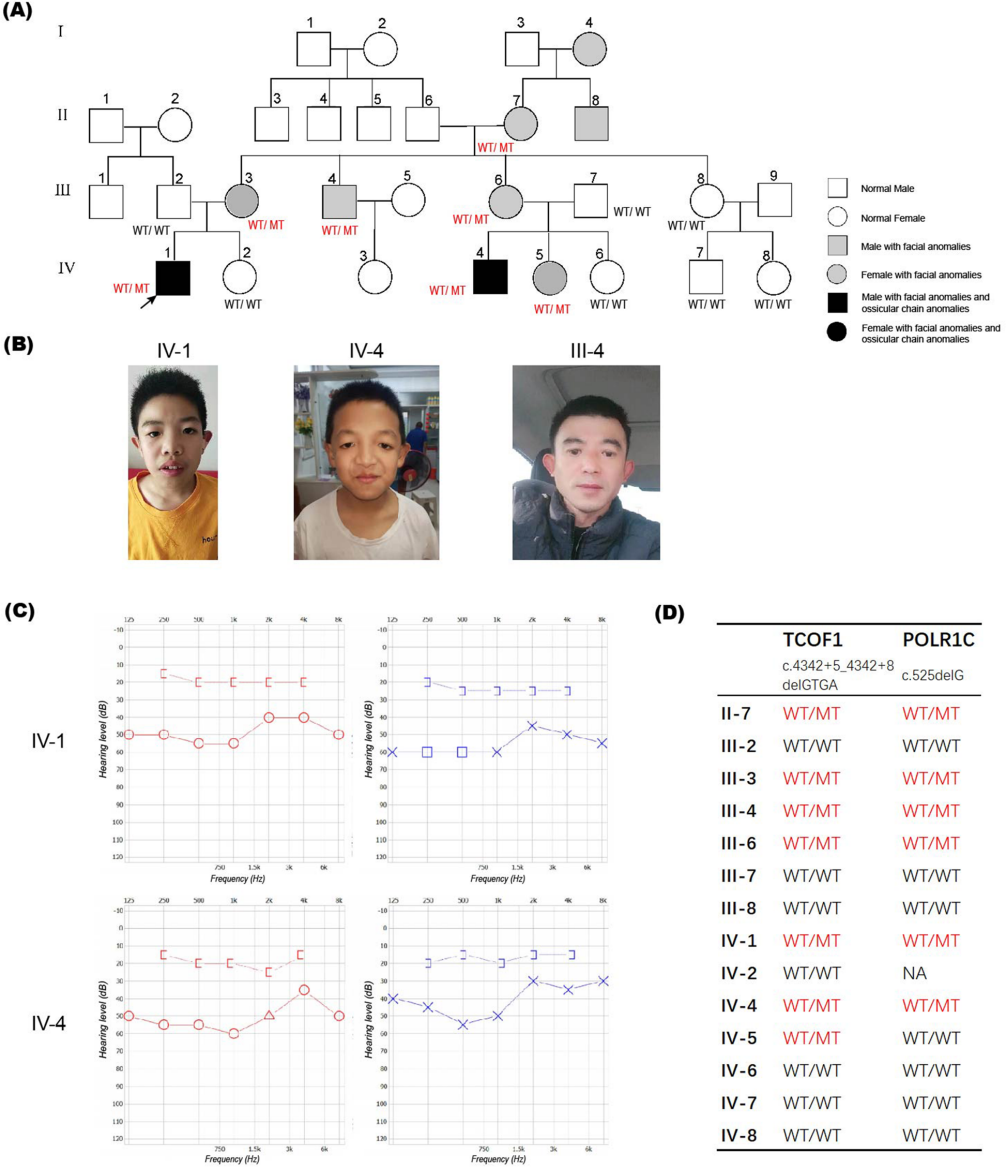
Clinical information and pedigree. **A** The pedigree of the family, including the proband (IV-1, arrow). Grey squares and circles indicate males and females, respectively, with facial anomalies but no conductive hearing loss. Black squares and circles indicate males and females, respectively, with facial and ossicular chain anomalies. The *TCOF1* c.4342 + 5_4342 + 8delGTGA mutational status of individuals available for genetic analysis is shown as WT/WT or WT/MT. **B** The proband and his cousin exhibited the typical facial phenotypes of Treacher Collins syndrome and their uncle with obvious but milder facial anomaly (III-4), i.e., overhanging lateral halves of the upper eyelids and downward slanting palpebral fissures. All individuals depicted herein gave written consent to the publication of the photographs. **C** Conductive hearing loss assessed via PTA of IV-1 and IV-4. Defects in air conduction were apparent; both ears had air-bone gaps. **D** Sanger sequencing of detected variants in family members revealed TCOF1: c.4342 + 5_4342 + 8delGTGA and POLR1C: c.525delG in family members. WT: wild type; MT: mutant; NA: not available

**Fig. 2:**
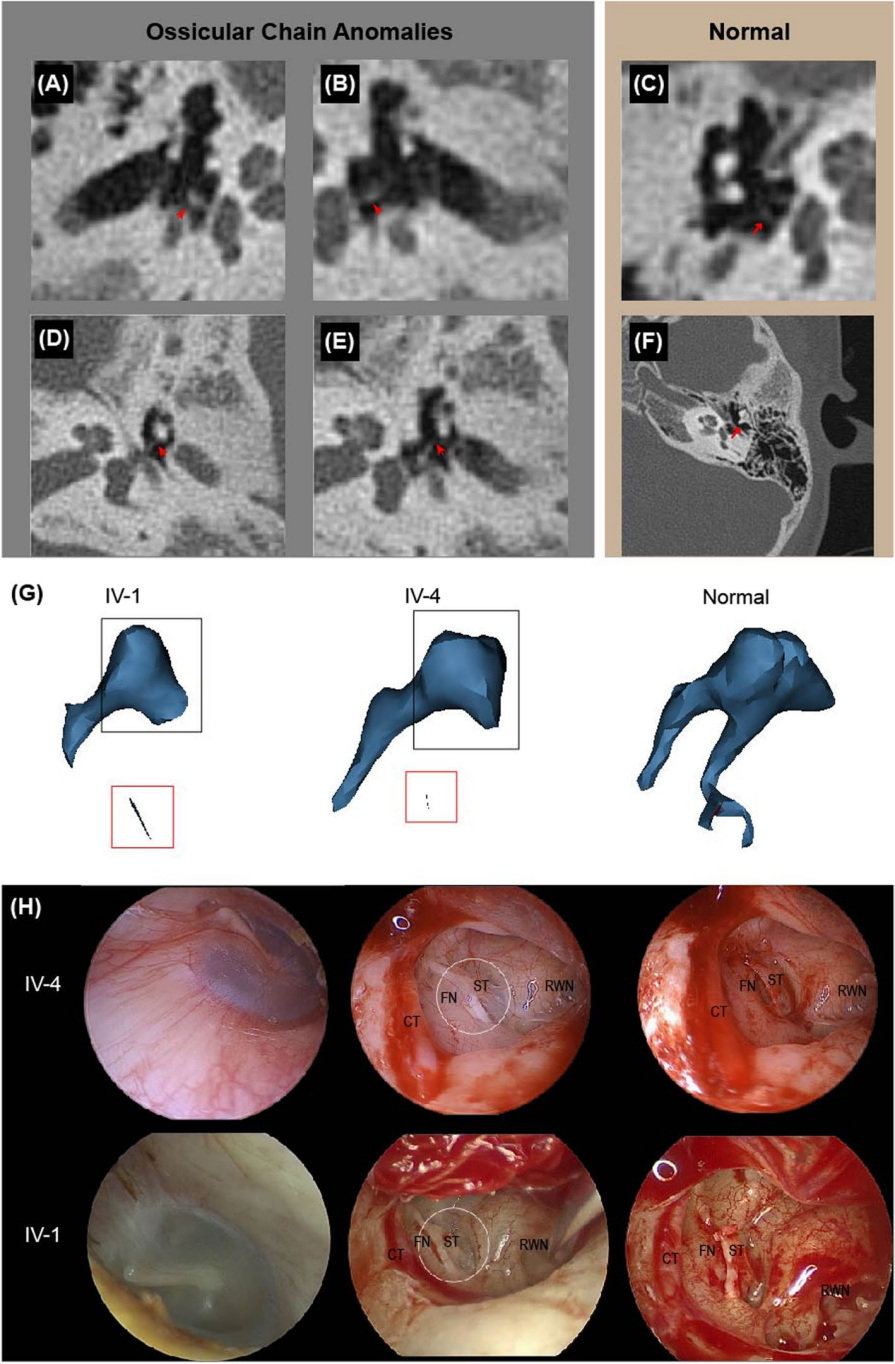
Ossicular chain malformations in IV-1 and IV-4. **A**-**F** CT scans of the TCS proband revealed ossicular chain anomalies. **A**, **B** Bilateral fusion of the anterior and posterior stapedial crura. **C** A normal stapes. **D**, **E** Malformation of the long crus of the incus. **F** A normal long crus of the incus. **G** Three-dimensional reconstruction of the ossicular chain of IV-1, IV-4 and normal person. Red block: stapes without footplate; Black block: malformation of incus **H** Intraoperatively, the ossicular chain anomalies in IV-1 and IV-4 were similar. CT: chorda tympani; FN: facial nerve; ST: stapes; RWN: round window niche

Proband IV-1 was an 11-year-old Chinese male who visited the hospital with hearing loss for at least 5 years; tinnitus, vertigo, nausea/vomiting, otalgia, and nasal discharge were not reported. On inspection, downward slanting palpebral fissures (Fig. 1A-B) and mandibular hypoplasia were observed. The proband’s cousin (IV-4) exhibited hearing loss and similar facial characteristics (Fig. 1C). After careful history-taking, seven family members with facial anomalies (mildly downward slanting palpebral fissures and mandibular hypoplasia) and normal hearing were noted (I-4, II-7, II-8, III-3, III-4, III-6 and IV-5). PTA of the proband and his cousin revealed bilateral conducive hearing loss with air-bone gaps on both sides (Fig. 1C). Temporal bone CT revealed malformation of the ossicles, with fusion of the anterior and posterior stapedial crura and bilateral fusion of the long crura of the incus and attic bony walls (Fig. 2A–G). These findings were further confirmed by intraoperative observations (Fig. 2H). Class III malformation was diagnosed using the criteria of Teunissen and Cremers [10] in both patients. The long crus of the incus was lacking, and a stape malformation and mobile stape footplate were apparent. The fused anterior and posterior stapedial crura formed thin plates near the tympanic segment of the facial nerve (Fig. 2H). Additional movie files of the three-dimensional reconstruction of the ossicular chain (without footplates) of IV-1, IV-4 and normal one show this in more detail (see Supplementary Video 1, 2, 3). TCS was suspected based on the typical clinical manifestations and the examinations. The family pedigree was consistent with an autosomal dominant inheritance pattern (Fig. 1A).

### Genetic analysis

**Fig. 3:**
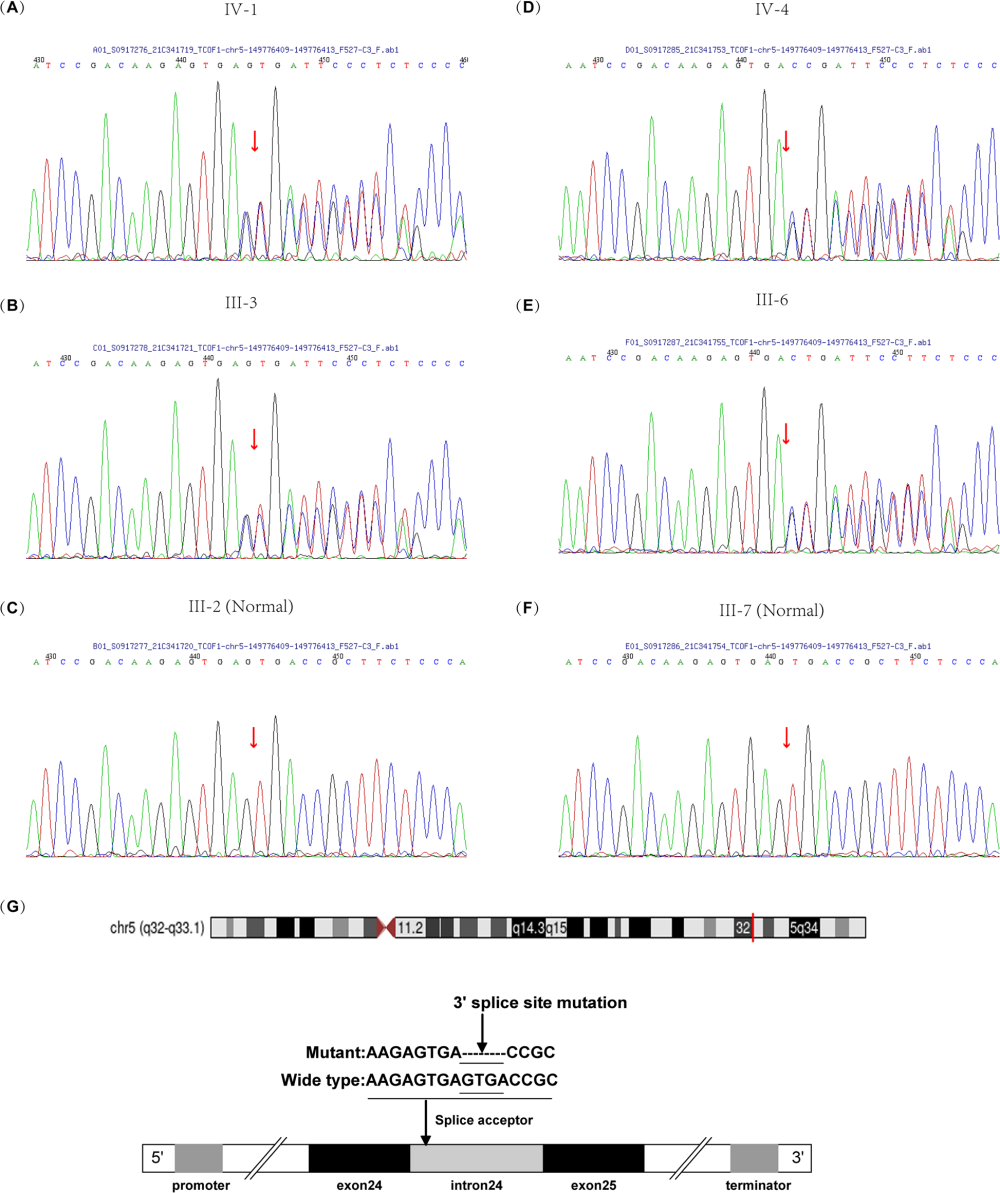
The suspected pathogenic variant. Sanger sequencing of *TCOF1* revealed a GTGA deletion in **A** the proband (IV-1) and **B** his mother (III-3), but not in **C** his father (III-2); and in **D** the cousin of the proband (IV-4) and **E** his mother (III-6), but not in **F** his father (III-7). **G** The location and pattern of the splice site variation in *TCOF1*

To search for potential pathogenic variants, we performed WES on IV-1, IV-4 and their parents. Average depth of coverage ranged from 234.66-fold to 380.25-fold. After filtering and segregation analysis, we identified a novel heterozygous variant c.4342 + 5_4342 + 8delGTGA in the *TCOF1* gene (NM_001371623.1), which was confirmed by Sanger sequencing (Fig. 3). This pathogenic variant was absent in multiple database (gnomAD, 1000 genome and in-house database). SpliceAI give a donor loss prediction of score 0.97 for this variant. Sanger sequencing of all available family members showed that the variant co-segregates with the phenotype (Fig. 1A, D). The variation is “Pathogenic” according to the ACMG variant guidelines (PM2_supporting + PP1_strong + PP4) as confirmed in vivo (Results 3.3) and in vitro (Results 3.4). Interestingly, a heterozygous variant in the *POLR1C* gene (NM_203290; exon6; c.525delG) was identified in both IV-1 and IV-4, which was also inherited from their mothers (Fig. S1). This variant was further tested in other family members in this pedigree (Fig. 1D). However, this variation is not currently considered pathogenic given the AR inheritance mode of TCS3.

### Splicing analysis

**Fig. 4:**
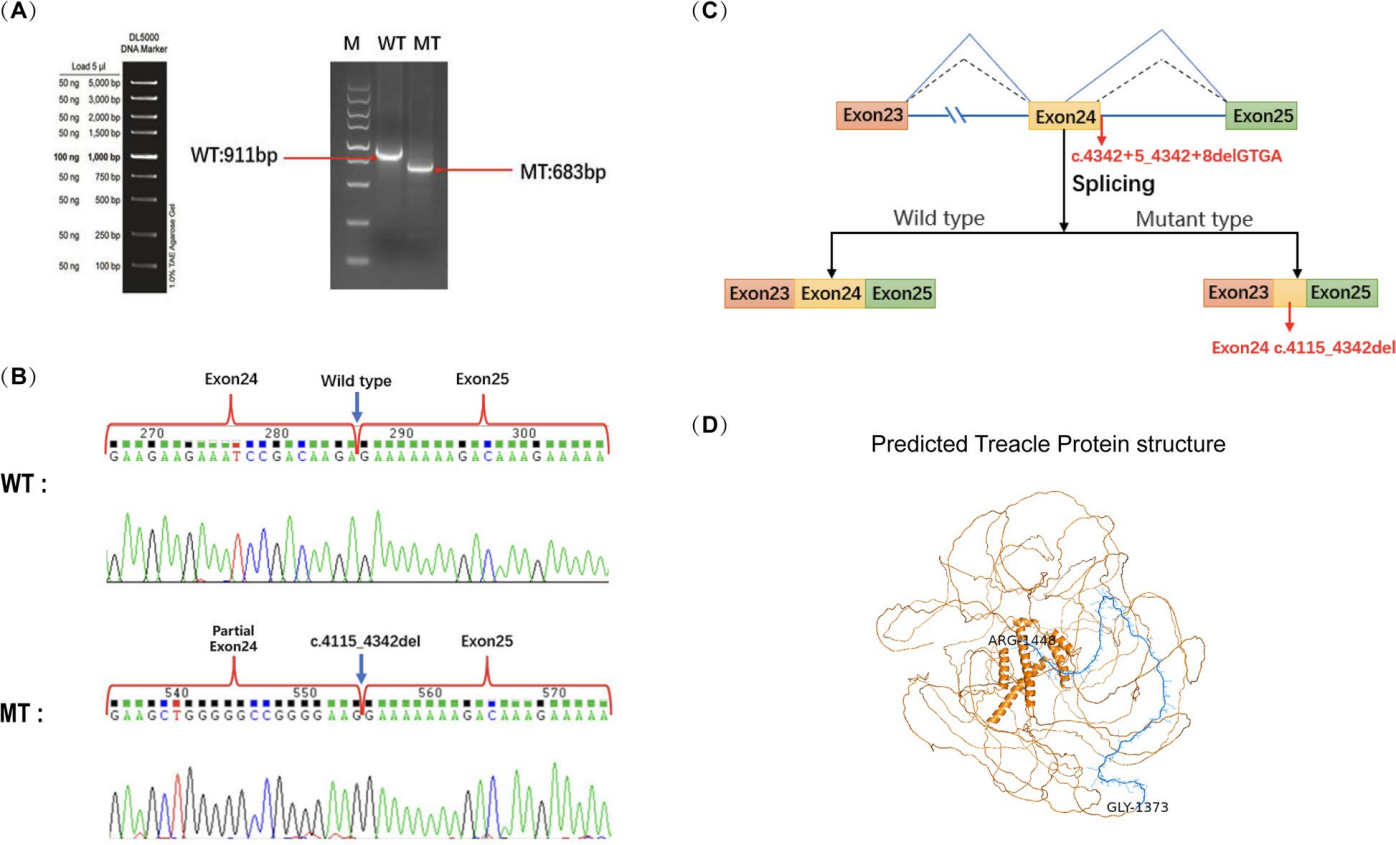
Minigene assay of the *TCOF1* c.4342 + 5_4342 + 8delGTGA splicing variant and a schematic of the splicing pattern. **A** RT-PCR of amplified WT and MT cDNA. **B** Sanger sequencing of the normal splicing isoform of the WT plasmid and isoform of the MT plasmid with c.4115_4342del. **C** Schematic of WT and MT splicing. **D** Predicted structure of the Treacle protein and the deleted amino acids p.Gly1373_Arg1448del (blue). WT, wild type; MT, mutant

**Fig. 5:**
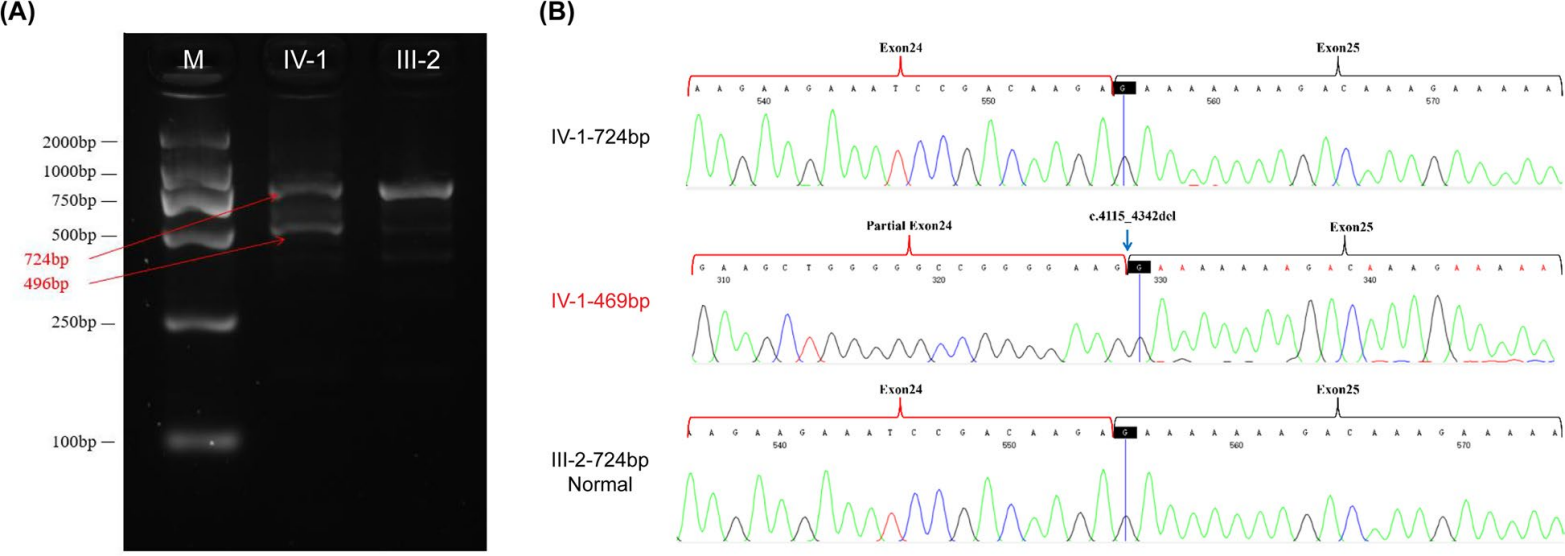
Confirmation of variant-induced spliceogenicity. **A** Identification of a short transcript in IV-1 with a 228-bp deletion downstream of exons 23–26 s attributable to the c.4342 + 5_4342 + 8delGTGA variation of exon 24. **B** Representative electropherograms of the pathogenic variant (IV-1-469 bp) and normal gene (724 bp)

To investigate whether the deletion affected splicing, minigene analysis was performed. Agarose gel electrophoresis of RT-PCR products showed a 911-bp band from wild-type cDNA and a 683-bp band from mutant DNA (Fig. 4A). Sanger sequencing revealed a normal splicing product from the wild-type allele, consistent with *TCOF1* exons 23–25. However, alignment showed that splicing product of the mutant lost 228-bps, suggesting that the pathogenic variant caused partial deletion of exon 24 which may yield a shortened protein (Fig. 4B-D). To confirm the splicing defects revealed by the minigene assay, we extracted RNA from peripheral blood of the patients and sequenced the mRNA of *TCOF1* (Fig. 5). For the proband, RT-PCR using primers encompassing exons 23–26 of *TCOF1* yielded two bands of size 724 and 496 bps. Meanwhile, the proband’s healthy father showed a single 724-bps band (Fig. 5A). Sanger sequencing of the PCR produce revealed that the abnormal splicing caused 228-bps deletion of exon 24, which was same as revealed by minigene assay (Fig. 5B).

## Discussion

*TCOF1* was the first gene associated with TCS, and accounted for 86% of all TCS cases; *POLR1D*, *POLR1C* and *POLR1B* accounted for 6%, 1.2%, and 1.3% of cases, respectively [5, 8, 11]. Over 200 causative variants have been reported but the molecular networks and underlying mechanisms remain elusive [10]. One systematic review of TCS found that of all *TCOF1* pathogenic variants, 66% were deletions, followed by substitutions (23%), insertions (6%), duplications (3%), and insertions/deletions (2%) [12].

The *TCOF1* gene contains 27 exons, among which 5 (exons 10, 15, 16, 23, and 24) bear approximately 50% of all pathogenic mutations [13]. *TCOF1* encodes a nuclear phosphoprotein (Treacle) that is 1,488 aa in length [14]. Mutations in *TCOF1* are usually family-specific and mostly produce truncated Treacle. Treacle is a spatiotemporal regulator of ribosome biogenesis during neural plate and crest formation [15, 16]; crest cells further differentiate into cartilage, bones, and connective tissues of the head and face. The reduced proliferative capacity of migrating cranial neural crest cells and neuroepithelial apoptosis, which is caused by the lack of mature ribosomes because of *TCOF1* haploinsufficiency during development, explains the characteristic craniofacial abnormalities of TCS [17]. Treacle is a nucleolar phosphoprotein with low N-terminal complexity, whereas the C-terminus (encoded by exons 17–26, with several nuclear localization signal [NLS] regions in exons 23–25 [18]) has 10 repeat units of serine clusters separated by alanine, lysine, proline, and glutamic acid [14, 19]. Several potential NLS regions lie between aa 1,362 and 1,482 in the C-terminal region [20].

In the present study, we report a heterozygous TCOF1 pathogenic variant c.4342 + 5_4342 + 8delGTGA in a Chinese pedigree characterized by ossicular chain malformation, downward slanting palpebral fissures, and mandibular hypoplasia evident to varying degrees among family members. As splicing assay showed that the pathogenic variant disrupted splicing and caused an in-frame deletion in exon 24, which may produce a mutate Treacle with shortened NLS domains. During the biogenesis of ribosomes in nucleoli, the C-terminus (aa 1,294–1,488) of Treacle interacts with UBF, Nopp140, and rDNA [21]. Loss of Treacle integrity (in terms of Gly1373_Arg1448) and low protein expression may inhibit rDNA transcription and thus compromise ribosome biogenesis and function. The splicing variant truncates Treacle protein and yields a typical clinical phenotype. We analyzed all the 355 pathogenic and likely pathogenic *TCOF1* variants in LOVD database, and only three splicing variations that lie outside the donor or acceptor splice sites involve the + 1/ + 2 or − 1/ − 2 position was identified. This implies that some non-canonical TCOF1 variants might be overlooked in the sequencing data.

As the clinical phenotypes of the heterozygous individuals in the family ranged from a slight facial abnormality to the typical TCS facial phenotype combined with ossicular chain malformation, gene expressivity varied, suggesting possible roles for disease-modifying factors. In this pedigree, severity was highest in males and the fourth generation of all heterozygous family members. Of all members in the first-to-third generations with facial anomalies, only III-4 (male) exhibited obvious malar and zygomatic hypoplasia. The females were almost normal. In the fourth generation, although IV-5 had a facial phenotype similar to that of III-4, i.e., much more severe than those of other WT/MT female family members, there were no ossicular chain anomalies. Conductive hearing loss was noticed at an early age in both males (IV-1 and IV-4), who were later diagnosed with ossicle malformations. Notably, the ossicular chain malformations were identical. Increased severity in successive generations is a common feature in familial TCS patients. Interestingly, a heterozygous *POLR1C* truncating variant c.525delG was found almost co-segregated with the *TCOF1* pathogenic variant (Fig. 1). Since *POLR1C* is inherited autosomal recessively, it was more likely to be a coincidence. Whether the variants from two TSC genes influence the phenotype interactively in this pedigree remains unkonwn. One genotype–phenotype correlation study found that the frequencies of mandibular hypoplasia (82.8%), malar hypoplasia (83.3%), and downward slanting palpebral fissures (88.4%) were significantly higher in patients with *TCOF1* variants, but conductive deafness was more common in patients with *POLR1* variants [12]. Among TCS patients with *POLR1C* variants, three out of four (75%) were males and they all presented with malar/zygomatic hypoplasia (100%), conductive deafness (100%), and mandibular hypoplasia/micrognathia (100%); three also exhibited downslanting palpebral fissures (75%) and coloboma of the lower lid (75%) [11, 22]. However, since only 7 variants in 4 families has been reported in *POLR1C* for TCS, sequencing data from more cases and molecular studies are required to analyze the interaction networks of TCS genes and their effect on phenotype.

## Conclusion

In conclusion, we identified a heterozygous *TCOF1* splicing variant c.4342 + 5_4342 + 8delGTGA (splicing) in a Chinese TSC family with ossicular chain malformations and facial anomalies. A more severe clinical phenotype with advancing generation, and in males, was noted in this four-generation family. Furthermore, a heterogenous *POLR1C* truncating variant was found almost co-segregating with the *TCOF1* pathogenic variant. Our findings broadened the spectrum of TCS variants and will facilitate diagnostics and prognostic predictions. Detection of more novel TCS pathogenic variant and phenotypes may reveal the underlying mechanisms and inheritance pattern of our pedigree family.

### Supplementary Information

**Supplementary Material 1.** 

**Supplementary Material 2.** 

**Supplementary Material 3.** 

**Supplementary Material 4.** 

**Supplementary Material 5.**

## Data Availability

The dataset supporting the conclusions of this article is included within the article.

## Abbreviations

TSC: Treacher Collins syndrome
RT-PCR: Reverse Transcription PCR
WES: Whole-exome sequencing
PTA: Pure tone audiometry
HRCT: High-resolution computed tomography
NLS: Nuclear localization signal
