# Supplementary material for: A novel intronic TCOF1 pathogenic variant in a Chinese family with Treacher Collins syndrome
Source: BMC Med Genomics. 2024 Mar 18;17:75. doi: 10.1186/s12920-024-01828-4 (PMC10946134; doi:10.1186/s12920-024-01828-4)
Supplement: Supplementary file 2 — Supplementary Material 2. [file 12920_2024_1828_MOESM2_ESM.docx]

**Supplementary materials**

**Supplemental table 1.** Subtypes of Treacher Collins syndrome (TCS).

AD, autosomal dominant; AR, autosomal recessive.


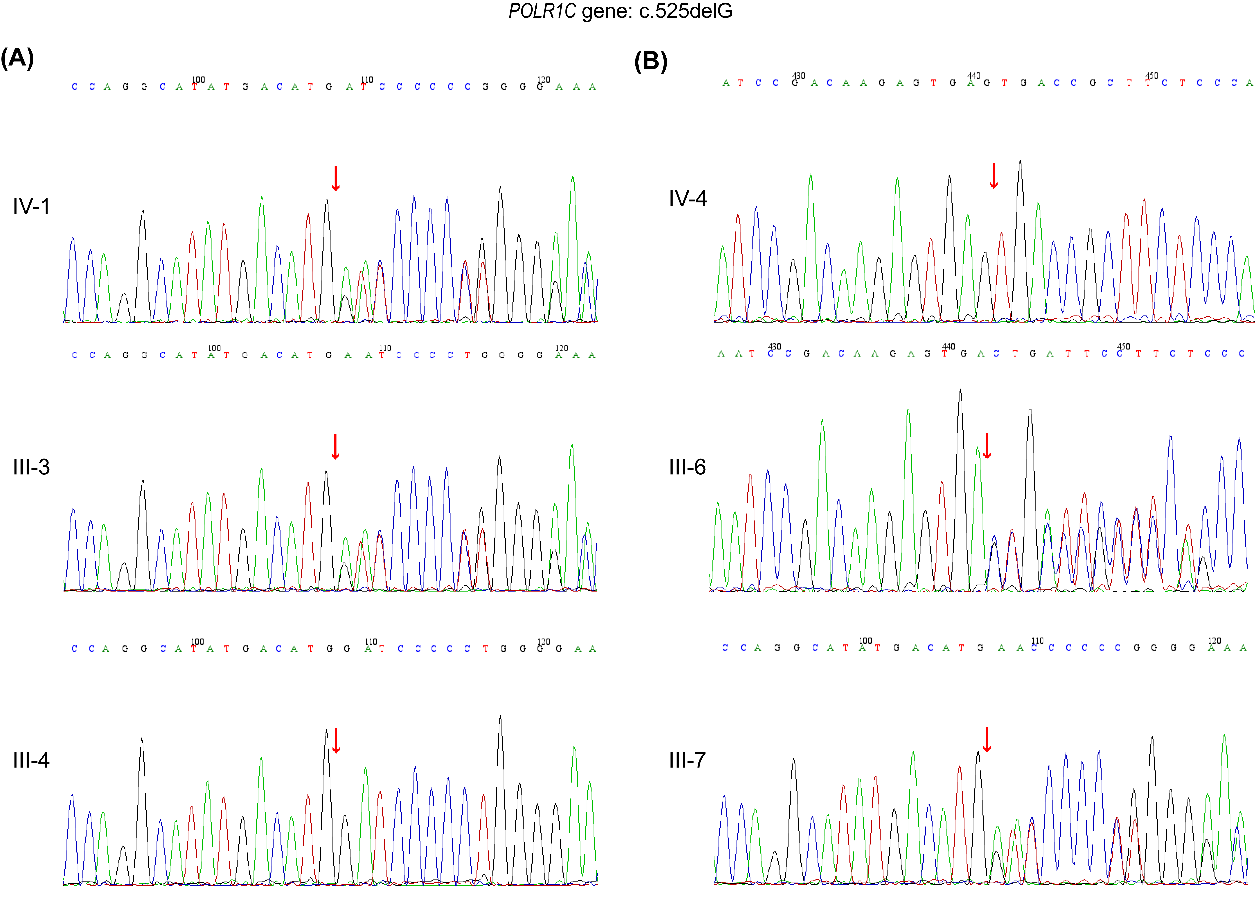


**Figure S1. Heterozygous mutation in *POLR1C* gene (NM_203290; exon6; c.525delG)** Sanger sequencing of *POLR1C* gene in both probands and their parents revealed a G deletion in IV-1, III-3, IV-4, III-6.

**Supplementary Video 1-3: Three-dimensional reconstruction of the ossicular chain anomalies**

**Video 1:** Three-dimensional reconstruction of the ossicular chain of IV-1. Shortened short crus of incus and missing long crus can be observed. Normal stapes was not visible, which is replaced by a thin plate.

**Video 2:** Three-dimensional reconstruction of the ossicular chain of IV-4. Shortened short crus of incus and missing long crus can be observed. Normal stapes was not visible, which is replaced by a thin plate.

**Video 3:** Three-dimensional reconstruction of normal ossicular chain with normal long and short crus of the incus, anterior and posterior stapedial crura

**Figure 5A Original image**


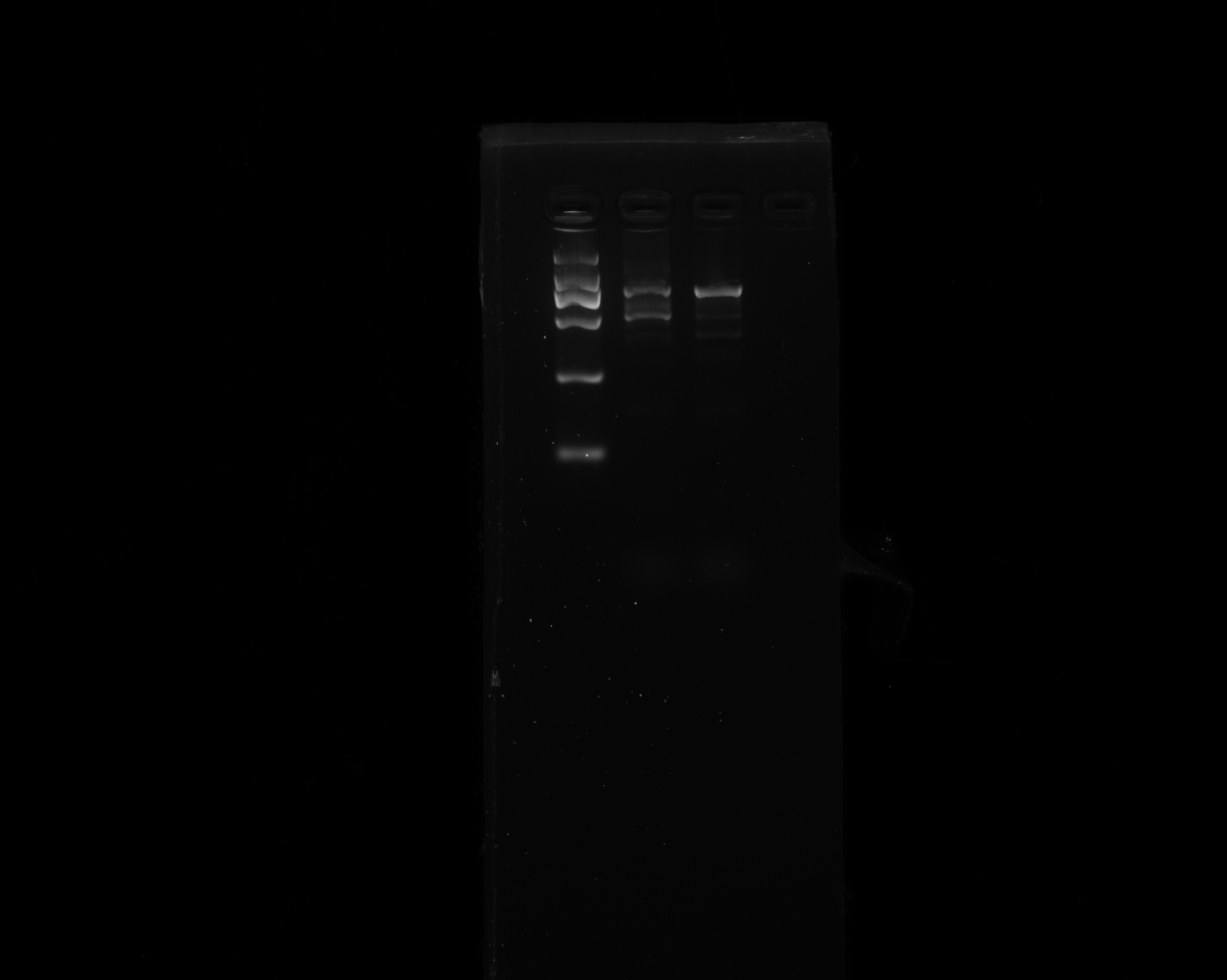


**Figure 4A Original image**


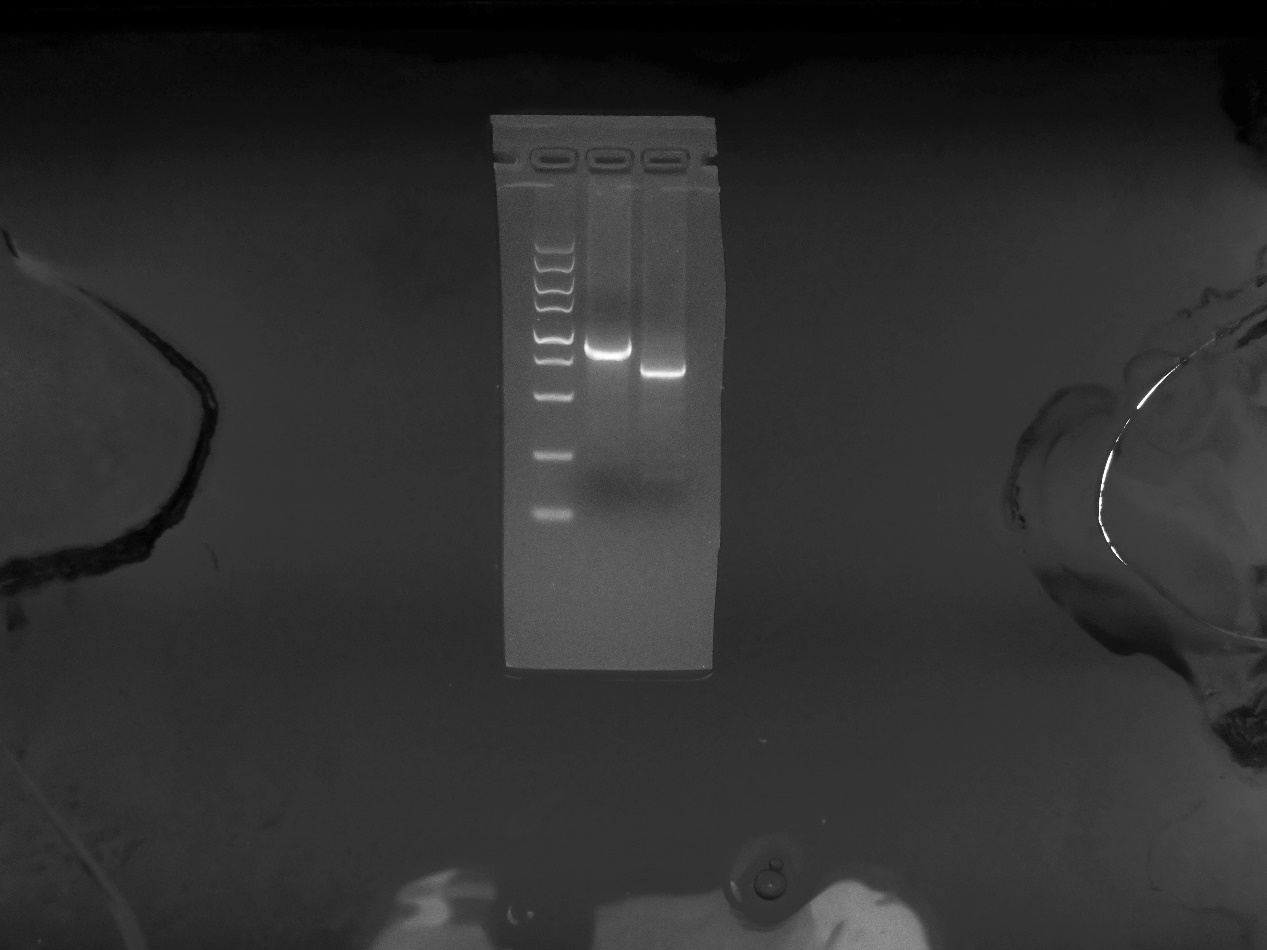
.
